# TU-Tagging: A Method for Identifying Layer-Enriched Neuronal Genes in Developing Mouse Visual Cortex

**DOI:** 10.1523/ENEURO.0181-17.2017

**Published:** 2017-10-04

**Authors:** Johanna Tomorsky, Leah DeBlander, Clifford G. Kentros, Chris Q. Doe, Cristopher M. Niell

**Affiliations:** 1 Institute of Neuroscience, University of Oregon, Eugene, OR 97403; 2 Institute of Molecular Biology, University of Oregon, Eugene, OR 97403; 3 Department of Biology, University of Oregon, Eugene, OR 97403; 4 Howard Hughes Medical Institute, University of Oregon, Eugene, OR 97403; 5 Kavli Institute of Systems Neuroscience, NTNU, Trondheim 7030, Norway

**Keywords:** gene expression, layer 2/3, postnatal, transcriptomics, TU-tagging, visual cortex

## Abstract

Thiouracil (TU)-tagging is an intersectional method for covalently labeling newly transcribed RNAs within specific cell types. Cell type specificity is generated through targeted transgenic expression of the enzyme uracil phosphoribosyl transferase (UPRT); temporal specificity is generated through a pulse of the modified uracil analog 4TU. This technique has been applied in mouse using a Cre-dependent UPRT transgene, *CA>GFPstop>HA-UPRT*, to profile RNAs in endothelial cells, but it remained untested whether 4TU can cross the blood-brain barrier (BBB) or whether this transgene can be used to purify neuronal RNAs. Here, we crossed the *CA>GFPstop>HA-UPRT* transgenic mouse to a *Sepw1-cre* line to express UPRT in layer 2/3 of visual cortex or to an *Nr5a1-cre* line to express UPRT in layer 4 of visual cortex. We purified thiol-tagged mRNA from both genotypes at postnatal day (P)12, as well as from wild-type (WT) mice not expressing UPRT (background control). We found that a comparison of Sepw1-purified RNA to WT or Nr5a1-purified RNA allowed us to identify genes enriched in layer 2/3 of visual cortex. Here, we show that Cre-dependent UPRT expression can be used to purify cell type-specific mRNA from the intact mouse brain and provide the first evidence that 4TU can cross the BBB to label RNA in vivo.

## Significance Statement

Visual cortex has a laminar organization with neurons in each layer having distinct functional characteristics and developmental timelines. Previous studies looking for layer-specific gene expression have examined embryonic, newborn, or adult cortex, while overlooking significant postnatal stages. Here, we used the transcriptional profiling technique, thiouracil (TU)-tagging, to isolate transcripts enriched in visual cortex layer 2/3 at postnatal day (P)12, a time of prolific synapse formation in this area. TU-tagging achieves spatial specificity via Cre-dependent expression of uracil phosphoribosyl transferase (UPRT), and temporal specificity via subcutaneous injection of 4TU. This study is the first to successfully use a transgenic mouse to apply TU-tagging to neuronal cell types and to demonstrate that 4TU can cross the blood-brain barrier (BBB) to tag neuronal RNA.

## Introduction

Quantifying patterns of gene expression during development or following exposure to different conditions, such as drug administration, can provide an understanding of genome function as it relates to underlying biological processes (Vandesompele et al., 2002; Cahoy et al., 2008; Wang et al., 2009; Gay et al., 2013). Unfortunately, many gene profiling techniques are limited by restricted access to specific cell types contained in complex tissues. The brain, which is the most complex mammalian organ containing cells with long-projecting delicate processes, presents a unique challenge when attempting to isolate cell type-specific transcripts. Although several techniques have been developed to characterize gene expression profiles in distinct cell types (Doyle et al., 2008; Sanz et al., 2009; Shapiro et al., 2013; Tallafuss et al., 2014; Poulin et al., 2016), most require either cell dissociation or tissue sectioning, which can damage neuronal projections (Shapiro et al., 2013; Tallafuss et al., 2014; Poulin et al., 2016).

Translating ribosome affinity purification (TRAP), RiboTag, and thiouracil (TU)-tagging are transcriptional profiling techniques that do not require cell isolation. TRAP and RiboTag can be used to identify cell type-specific translation of RNA by immunoprecipitating, from whole tissue homogenates, mRNAs attached to 80s ribosomes either HA-epitope tagged (RiboTag) or fluorescent reporter-tagged (TRAP) in distinct cell types (Doyle et al., 2008; Sanz et al., 2009). The TU-tagging technique, on the other hand, utilizes cell type-specific expression of the enzyme uracil phosphoribosyl transferase (UPRT) to identify genes actively transcribed in those cell types (Gay et al., 2013, 2014). UPRT works to convert injected 4TU to 4-thiouridine, which is incorporated into newly transcribed RNA. Thiol-tagged RNA can later be purified from whole tissue homogenates and subjected to high-throughput Illumina sequencing (Gay et al., 2013, 2014). Unlike RiboTag and TRAP, TU-tagging can be used to identify RNAs that may not be actively translated or ribosome associated, and can therefore provide a broader picture of cell type-specific gene expression. Here, we applied the TU-tagging technique in mouse brain to identify genes expressed in upper layer neurons of the developing visual cortex.

Mouse visual cortex is organized in layers with distinct functional properties and unique timelines for the development of these properties (Hoy and Niell, 2015). Neurons in each layer of the cortical circuit need to find the correct synaptic partners during development for proper processing of visual information. Eye opening, which occurs between postnatal days (P)12 and P14 represents a peak of synapse formation in the visual cortex, and is known to be a time of dynamic gene expression, which could be generating-specific patterns of connectivity (Yoshii et al., 2011). Transcriptional profiling studies of layer-specific gene expression in visual cortex often focus on adult or embryonic and newborn developmental time points, leaving the developmental stage around eye opening largely neglected (Belgard et al., 2011, Molyneaux et al., 2015; Poulin et al., 2016). Here, we used a modified TU-tagging protocol similar to that used in Chatzi et al. (2016) to profile neuronal RNA from visual cortex layer 2/3 at P12, a time point just before eye opening.

The TU-tagging technique was previously applied in mouse using a published UPRT transgene (Gay et al., 2013) to profile murine endothelial RNAs, and recently using viral injection to express UPRT in newly generated dentate granule neurons (Gay et al., 2013; Chatzi et al., 2016). Here, we are the first to successfully apply TU-tagging in mouse neurons using a transgenic mouse, making the method more accessible to those wishing to isolate cell type-specific mRNA from the mammalian brain. Through this study, we identified genes with expression enriched in visual cortex layer 2/3 at P12, while also providing evidence that 4TU can cross the blood-brain barrier (BBB).

## Materials and Methods

All experimental protocols were approved by the University of Oregon Institutional Animal Care and Use Committees, in compliance with the National Institutes of Health guidelines for the care and use of experimental animals.

### Layer-specific expression of UPRT and tissue dissection

Homozygous *CA>GFPstop>HA-UPRT* mice (Gay et al., 2013) were crossed with *Sepw1-cre* or *Nr5a1-cre* transgenic lines to achieve cortical layer-specific expression of the UPRT enzyme. Wild-type (WT) mice were processed identically to Cre-positive mice to produce the WT-pure sample type. The visual cortexes from four mice were required per sample to produce enough starting material for biotin-streptavidin purification of tagged RNA. Therefore, only litters with at least four Cre-positive pups were used (each sample is a mix of genders). All samples were collected at P12. A total of 50 mg of 4TU (Sigma-Aldrich) was dissolved in 250 μl of DMSO for injection. Mice were injected with 4TU (430 mg/kg in DMSO) in the morning, and visual cortexes were collected 5-6 h later. Dissection of visual cortex was performed in RNAlater (Thermo Fisher Scientific). Both left and right visual cortexes were stereotaxically marked with DiD fluorescent dye (2.5 mm from the midline and 1 mm from the back suture), and a ∼1-mm^2^ section of cortex was cut around the mark. Visual cortex samples were frozen in RNAlater (per manufacturer’s instructions) at −80°C until RNA extraction and purification.

## Immunohistochemistry

**Figure 1.**
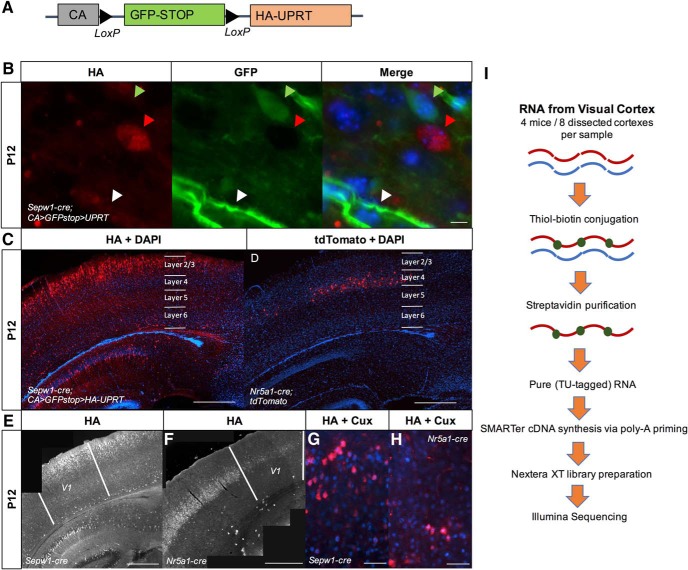
Upper cortical layer-enriched neuronal expression of HA-UPRT. ***A***, Diagram of the *CA>GFPstop>HA-UPRT* transgene (Gay et al., 2014). ***B***, Expression of the *CA>GFPstop>HA-UPRT* transgene (Gay et al., 2013) crossed to a *Sepw1-cre* line in neurons and endothelial cells (40× objective, scale bar: 10 μm). Green arrow: GFP-positive, Cre-negative neuron; white arrow: GFP-positive, Cre-negative endothelial cell; red arrow: UPRT:HA-positive, Cre-positive neuron. ***C***, *Sepw1-cre* drives UPRT expression in layer 2/3 and to a lesser extent layer 4. Immunostaining for HA at P12 in a *Sepw1-cre; CA>GFPstop>HA-UPRT* cross reveals layer 2/3-enriched expression (10× objective, scale bar: 500 μm). DAPI is included to show cortical structure. ***D***, *Nr5a1-cre* drives expression in a sparse subset of layer 4 neurons. *Nr5a1-cre* crossed to a tdTomato marker at P12 is specific to layer 4 (10 objective, scale bar: 500 μm). DAPI is included to show cortical structure. ***E***, ***F***, UPRT: HA is expressed in visual cortex of *Sepw1-cre* (***E***) or *Nr5a1-cre* (***F***) mouse lines crossed to the *CA>GFPstop>HA-UPRT* line (5× objective, scale bar: 500 μm). ***G***, ***H***, *Sepw1-cre* and *Nr5a1-cre* drive expression in neurons found predominantly in layer 2/3 or layer 4 of visual cortex, respectively. Neurons immunostained for the HA epitope on UPRT (red) in *Sepw1-cre; CA>GFPstop>HA-UPRT* or *Nr5a1-cre; CA>GFPstop>HA-UPRT* crosses, express the upper layer neuronal marker Cux1 (blue) (40X objective, scale bar: 50 μm). ***I***, TU-tagging workflow using poly-A priming for cDNA synthesis and Nextera XT for library preparation.

Immunohistochemistry was performed on double transgenic mice at P12 to confirm HA-UPRT expression in cortical layer-specific cell types (Fig. 1). Mice were perfused and brains were extracted at P12 and fixed overnight at room temperature in 4% PFA (in 1× PBS). Brains were then transferred to a 30% sucrose solution (in 1× PBS) and either kept overnight at room-temperature or for 48 h at 4°C. Brains were then cryosectioned onto Superfrost Plus slides (Fisherbrand), and frozen at -80°C for long-term storage. To stain, slides were removed from the freezer and treated for 5 min with 300-μl 0.05% trypsin (Thermo Fisher Scientific; Hoy et al., 2013). Slides were then washed twice for 10 min with PBS and then once for 10 min with PBT (0.3% Triton X-100 in PBS). A blocking solution of 5% goat and 5% donkey serum in PBT was then applied to the slides and allowed to incubate at room temperature for 1-3 h. Slides were then drained and a solution of 2 μl/ml of HA-mouse (Covance Research Products catalog MMS-101P, RRID:AB_2314672) and 3 μl/ml anti-GFP chicken (Aves Labs catalog GFP-1020 RRID:AB_10000240) primary antibodies in block was applied. Slides were stored with primary antibody at 4°C overnight. The next day, slides were washed four times for 10 min each in PBT. A total of 4 μl/ml of secondary antibodies, mouse-555 (Thermo Fisher Scientific catalog A-21424 RRID:AB_2535845) and chick-488 (Jackson ImmunoResearch catalog 703-545-155 RRID:AB_2340375), in PBT was then applied to the slides and either kept overnight at 4°C or for 3 h at room temperature. Slides were then subjected to four more 10-min washes, two in PBT and two in PBS. A total of 2 μl/ml of 4’, 6-diamidino-2-phenylindole (DAPI) in PBS was then applied to slides for 5 min, after which slides were dried and mounted with VECTASHIELD mounting media (Vector Labs).

### RNA processing and preparation for sequencing

To obtain thiol-tagged RNA from layer 2/3 neurons in visual cortex, we used a modified TU-tagging protocol similar to that published in Chatzi et al. (2016). RNA was extracted by grinding tissue from mouse visual cortex into 1 ml of TRIzol (Thermo Fisher Scientific). Ground tissue in TRIzol stood at room temperature for 5 min before the addition of 200 μl of chloroform. The chloroform-TRIzol mix was vortexed for 15 s and allowed to separate for 2–3 min at room temperature before centrifuging at 4°C at 12,000 rpm for 15 min. The upper aqueous layer was kept, an equal volume of 70% ethanol was added, and the RNA was then purified on columns as per manufacturer’s protocol (PureLink RNA Minikit, Ambion). The quality of all RNA samples was analyzed on an Agilent Bioanalyzer (2100), and only samples with RNA integrity numbers (RINs) >8.0 were subsequently streptavidin purified. RNA was then biotinylated (10 μl 10× TE and 25 μl of 1 mg/ml EZ-link Biotin-HPDP in dimethylformamide) and streptavidin purified (uMACS Streptavidin kit) as previously described (Gay et al., 2014).

RNA concentrations were determined via qubit fluorometric quantitation or using the Agilent Bioanalyzer (2100) before cDNA preparation. To preserve RNA quality and quantity, a previously described fragmentation step was removed from this modified workflow, and poly-A priming was used for cDNA synthesis (SMARTer Ultra Low Input RNA kit for Sequencing, v3) instead of random priming after Ribo-Zero rRNA removal (Fig. 1*D*; Gay et al., 2014; Chatzi et al., 2016). A total of 5-10 ng of RNA was used for cDNA preparation with the SMARTer kit. Library preparation was performed on 1 ng of cDNA using Illumina’s Nextera XT Library Preparation kit. Samples were then pooled in groups of six and subjected to 100-bp single-end sequencing on an Illumina HiSeq instrument (Fig. 1*D*).

## Sequence processing and differential expression analysis

All sequences were first filtered to remove reads that did not pass Illumina’s chastity filter. FastQC reports were then produced to identify overrepresented sequences and other quality concerns (Andrews, 2014). Overrepresented sequences (SMARTer adapter and Nextera primers) were then removed using the CutAdapt python package (Martin, 2011). The java program Trimmomatic was used to trim sequences based on quality (Bolger et al., 2014). Quality-trimmed sequences were then aligned to the mouse genome assembly GRCm38 (downloaded off the Ensembl browser) using the Genomic Short-read Nucleotide Alignment Program (GSNAP; Wu and Nacu, 2010; Cunningham et al., 2014). Finally, sequences that aligned to a particular gene were counted using the python program htseq-count in intersection-strict mode (Anders et al., 2014). The final gene counts were further filtered to isolate protein coding genes (identified using Ensembl-BioMart; Cunningham et al., 2014) for future analysis (raw gene counts for all samples are listed in Table 1-4). The DESeq package (version 1.24.0; Anders and Huber, 2010, 2012) was used to analyze differential expression between sample types defined as streptavidin purified RNA from (1) a *Sepw1-cre; CA>GFPstop>HA-UPRT* cross (Sepw1-pure), (2) an *Nr5a1-cre; CA>GFPstop>HA-UPRT* cross (Nr5a1-pure), or (3) WT mice not expressing UPRT (WT-pure). Pairwise comparisons were performed between Sepw1-pure and Nr5a1-pure sample types, and Sepw1-pure and WT-pure sample types, and a DESeq adjusted *p* value (Benjamini-Hochberg adjusted for multiple testing) of 0.1 was used as a cutoff to determine enrichment (Anders and Huber, 2010). Transcripts with few reads (three or more samples containing fewer than 1 count per million) were removed before DESeq analysis.

The functional categories representing Sepw1-enriched genes was examined through gene ontology (GO) analysis using GO-TermFinder (Boyle et al., 2004; go.princeton.edu) with a *p* value cutoff of 0.01 and MGI (*Mus musculus*) annotation. GO-enriched categories were then input to REVIGO (http://revigo.irb.hr/; Supek et al., 2011), an online tool used to summarize GO results by reducing redundant GO terms and finding broader representative categories for collections of genes (Fig. 4). The layer specificity of Sepw1-enriched genes was investigated by comparing DESeq gene enrichments to layer-enriched genes (400 genes with the highest probability of enrichment in each cortical layer from the online database described in Belgard et al., 2011), and examining Allen Brain Atlas developing mouse brain *in situ* data at P14 (Allen Brain Atlas 2008; Sepw1-Nr5a1 comparison). Expression patterns observed in Allan Brain in situs were classified manually as either “enriched” (reasonable observer would identify expression as darkest in layer 2/3 visual cortex), “present” (clear expression in layer 2/3 visual cortex, but not darkest here), or “depleted” (expression is not seen or very light in 2/3, and is dark in other areas). In situs that were unclear, were excluded from the analysis. In addition, in house *in situ* hybridizations were performed for seven Sepw1-enriched genes (Nr5a1-pure comparison) at P12.

### *In situ* hybridization

Nonradioactive colorimetric RNA *in situ* to quantify gene expression patterns was performed as previously described (Lein et al., 2007; Wehr et al., 2009). Briefly, tissue was prepared and sectioned as described for immunohistochemistry, after which 30-µm sections were brought to room temperature, washed three times 30 min each in 1× PBS, and then acetylated for 10 min in a 0.25% acetic anhydride solution in 0.1 M triethanolamine HCl (Lein et al., 2007). Slides were then covered with hybridization solution (50% formamide, 10% dextran sulfate, 1× Denhardt’s solution, 1 mg/ml yeast tRNA, 5× SSC, 0.1% Tween 20, and 0.1 mg/ml heparin in DEPC-treated H_2_O), fitted with a coverslip, and prehybridized for 2 h in a humidity chamber at 62°C or 70°C depending on the probe. To visualize transgene expression in the newly developed *TetO-UPRT* mouse (see below), a digoxygenin-labeled riboprobe was used, targeting 302 bp of the wood chuck picornavirus response element (WPRE), diluted 1:500 in hybridization solution. This riboprobe was generated using T3 RNA polymerase in the presence of dig-labeled nucleotides using the pBSKS-WPRE construct linearized with Nco1 as template. The riboprobes to *Gad*, *Sez6l2*, *Speg*, *Frmpd4*, *Tspan6*, *Pvrl3*, *Rgs8*, and *Pvrl1*, were diluted to a final concentration of 1–2 ng/μl in hybridization solution and were generated using the SP6 RNA polymerase in the presence of dig-labeled nucleotides, using probe sequences and protocols described by the Allen Brain Institute (Allen Institute for Brain Science, 2004; Lein, 2007; Allen Institute for Brain Science, 2008).

Slides were hybridized with each probe in hybridization solution overnight at 62°C for the WPRE probe and 70°C for all other probes. Sections were then washed three times for 30 min each in wash buffer (50% formamide, 0.5× SSC, 0.1% Tween 20) at the same temperature used for hybridization. Slides were then washed an additional three times for 30 min each at room temperature in MABT (1× maleic acid, 20% Tween 20) and then incubated in blocking solution (MABT, 20% sheep serum, 2% blocking reagent Roche number 11096176001) for 3 h. Anti-dig sheep Fab fragments conjugated to alkaline phosphatase (Roche number 11093274910) diluted 1:2500 in blocking solution were then added, and the slices were incubated at 4°C overnight. Slices were then washed at room temperature with MABT buffer five times for 5 min each and then AP staining buffer (0.1 M NaCl, 50 mM MgCl_2_, 10% polyvinyl alcohol 100,000–150,000 MW, and 0.1 M Tris-HCl, pH 9.5), twice for 10 min each, after which 3.5 μl/ml NBT, 2.6 μl/ml BCIP, and 80 μl/ml levamisole were added. The colorimetric reaction was allowed to develop for 3–48 h at 37°C and stopped by washing twice with PBS (0.1% Tween 20) and twice in deionized H_2_O. Slides were then either dehydrated in graded ethanols and mounted with Permount, or double-labeled via immunohistochemistry as described above.

## Microscopy

RNA *in situs* to WPRE were viewed on an Olympus BX61 wide field epifluorescence microscope with Prior ProScanIII motorized stage and Lumen 200 mercury lamp. Images were acquired using an Olympus DP72 12.8 megapixel camera and a 10× objective (UPlanApo 0.4 numerical aperture). Whole-slice composites were generated automatically using MetaMorph premiere software.

All other in situs and immunohistochemistry were viewed on a Zeiss Axio Imager.A2 wide field epifluorescence microscope with an X-Cite 120Q LED excitation lamp. Images were acquired with a Zeiss AxioCam MRm 1.4 megapixel camera and EC Plan-NEOFLUAR 5x/0.16 or EC Plan-NEOFLUAR 40x/0.75 objectives. Images were viewed using ZEN lite imaging software (2012) and in silico background removal and color processing of images were performed using Adobe Photoshop CS6.

## Statistical analysis

A resampling approach was used to determine if the amount of overlap seen between Sepw1-enriched genes and layer-enriched database genes was significantly greater than what might occur by chance (Table 1-1). To accomplish this, a short program was written in R to randomly sample the same number of genes as was enriched in each experimental comparison (Sepw1-pure to WT-pure, 1907 Sepw1-enriched; Sepw1-pure to Nr5a1-pure, 634 Sepw1-enriched) from the filtered gene counts for that particular comparison, and then determine to what extent this random subset overlapped with database layer-enriched genes. This program was looped to repeat this random sampling and determination of overlap 1000 times to produce a resampling distribution with an estimate of the mean and 95% confidence intervals; *p* values were calculated using the equation: (sum(resampled values < (estimate – distance from experimental value)) + sum(resampled values > (estimate + distance from experimental value)))/1000). It was necessary to repeat this with each database list of layer-enriched genes, since a different number of database genes from each of these categories were present in the “filtered counts” list of genes for each comparison (genes not in filtered counts are excluded from DESeq differential expression determination).

Binomial logistic regression analysis was performed using the glm function in R, and plotted to show the predicted likelihood of finding layer 2/3 genes as a function of the rank of Sepw1-fold enrichment.

### Development of the *TetO-UPRT* mouse

WPRE and SV40 intron sequence elements were amplified from a stock vector using primers containing EcoRV and XbaI restriction enzyme sites. The forward primer contained the EcoRV site: 5’TTTTTTGATATCTTGGTCCTGCTGGAGTTCGTGA, and the reverse primer contained the XbaI site: 5’AAAAAATCTAGAAACAGATGGCTGGCAACTAGAAG. After amplification, the WPRE-SV40 fragment and a pTRE-tight2 vector (containing the tetracycline responsive element and an SV40 poly A signal) were digested using aforementioned restriction enzymes and ligated together. The newly ligated vector was then used to transform *Escherichia coli* and a single positive clone was selected for further amplification and purification using a PureYield plasmid Midiprep kit (Promega). The resulting pTRE-tight 2 vector containing WPRE-SV40 (subsequently referred to as pTT-WPRE) was sequenced using a primer to the SV40 intron to confirm sequence fidelity. To add UPRT to the pTT-WPRE vector, stock pBSSK(+)-UPRT (used for the original development of the *CA>GFPstop>HA-UPRT* transgene Gay et al., 2013) was amplified and purified using the PureYield plasmid Midiprep kit (Promega). Purified pBSSK(+)-UPRT and pTT-WPRE were then digested overnight using restriction enzymes Not1 and Sal1 (the SV40 intron was removed with this step), ligated together, and used to transform *E. coli* overnight. Positive clones were selected by restriction enzyme screening and vector fidelity was confirmed by sequencing. The final vector was purified (PureYield plasmid Midiprep kit) then digested overnight with Nsp1 to linearize and isolate the entire tetO-UPRT-WPRE-SV40 construct. The final construct was purified using a gel-extraction kit (QIAGEN), and eluted in filtered microinjection buffer (low TE, pH 8.0) for pronuclear injection. Mice were genotyped to establish successful integration of the construct using primers to the WPRE element: 5’TCTCTTTATGAGGAGTTGTGGCCC, and 5’CGACAACACCACGGAATTGTCAGT. The resulting founder mice were crossed to a *CaMKII-tTA* line (The Jackson Laboratory) and screened for high levels of neuronal expression.

## Results

### Generating UPRT expression and purifying RNA enriched in upper layer cortical neurons in the postnatal brain

To determine if the *CA>GFPstop>HA-UPRT* mouse line (Fig. 1*A*; Gay et al., 2013, 2014) could be used to express UPRT in postnatal mouse neurons, we first crossed this mouse to *Sepw1(NP39)-cre*, a Cre driver line produced by the GENSAT project (Gerfen et al., 2013) with expression previously described, and confirmed here, to be enriched in layer 2/3 cortical pyramidal neurons (Fig. 1*C*,*E*,*G*). In this experiment, all Cre-negative cells should be GFP-positive and UPRT:HA-negative whereas all Cre-positive cells should be UPRT:HA-positive and GFP-negative (Fig. 1*B*). We found that in P12 visual cortex, *Sepw1-cre* generated UPRT expression that was enriched in layer 2/3 (Fig. 1*C*,*E*,*G*). We concluded that the *Sepw1-cre* line could be used to express UPRT in layer 2/3 of P12 mouse visual cortex.

After confirmation of UPRT expression, *Sepw1-cre; CA>GFPstop>HA-UPRT* double transgenic mice were used to thiol-tag and purify RNA from layer 2/3 cortical neurons, subsequently called “Sepw1-pure” RNA (Fig. 1*H*). Briefly, we injected 4TU subcutaneously into mice double positive for the *Sepw1-cre* and *CA>GFPstop>HA-UPRT* transgenes at P12, and 5–6 h later, dissected out the left and right visual cortexes. Each sample contained a pool of visual cortexes from 4 mice of mixed genders to provide enough material for subsequent purification. RNA was extracted, biotinylated, and streptavidin purified to produce samples enriched with thiol-tagged transcripts. All samples were then prepared for Illumina sequencing (Fig. 1*I*; Materials and Methods).

We prepared two different sample types as comparisons to find genes enriched in the Sepw1-pure samples. WT mice lacking the UPRT transgene were processed and streptavidin purified identically to the mice expressing UPRT in upper layer cortical neurons to approximate “background” from unlabeled and mislabeled RNAs (subsequently referred to as WT-pure). In addition, we used identically processed, thiol-tagged RNA from *CA>GFPstop>HA-UPRT* mice crossed to an *Nr5a1-cre* line, which we and others have demonstrated labels neurons in layer 4 of visual cortex (Harris et al., 2014; Oh et al., 2014) (subsequently referred to as Nr5a1-pure; Fig. 1*D*,*F*,*H*). We found it was important to only compare sample types that experienced a similar processing pipeline, i.e. subjected to the same purification procedure. For this reason, we avoided comparing purified RNA to “total” unpurified RNA samples, as was done in the first published TU-tagging protocols (Gay et al., 2013, 2014).

### DESeq differential expression analysis reveals transcripts enriched in layer 2/3

To determine novel genes enriched in layer 2/3 of developing visual cortex, Sepw1-pure, WT-pure, and Nr5a1-pure RNA sample types were sequenced to a depth ranging from 21–38 million reads (SRA accession number: *SRP097635*). FastQC reports demonstrated high sequence quality (average per base sequence quality > 30 at all positions) and low duplication rates (>70% remaining after deduplicaton) for all samples after trimming and filtering sequences. Over 90% of reads for all samples uniquely mapped to the mouse genome (Cunningham et al., 2014) using the splice-aware GSNAP (Wu and Nacu, 2010). Mapped sequences that aligned to particular genes were then counted using the python program htseq-count, filtered to remove genes with low reads (Robinson et al., 2010), and normalized to counts per million using the DESeq package (version 1.24.0) in R (Anders and Huber, 2010, 2012). Removing low-count transcripts reduced the total number of genes analyzed by DESeq from 22,078 (entire mouse transcriptome) to 13,849 for the Sepw1-Nr5a1 comparison or 13,891 for the Sepw1-WT comparison.

**Figure 2.**
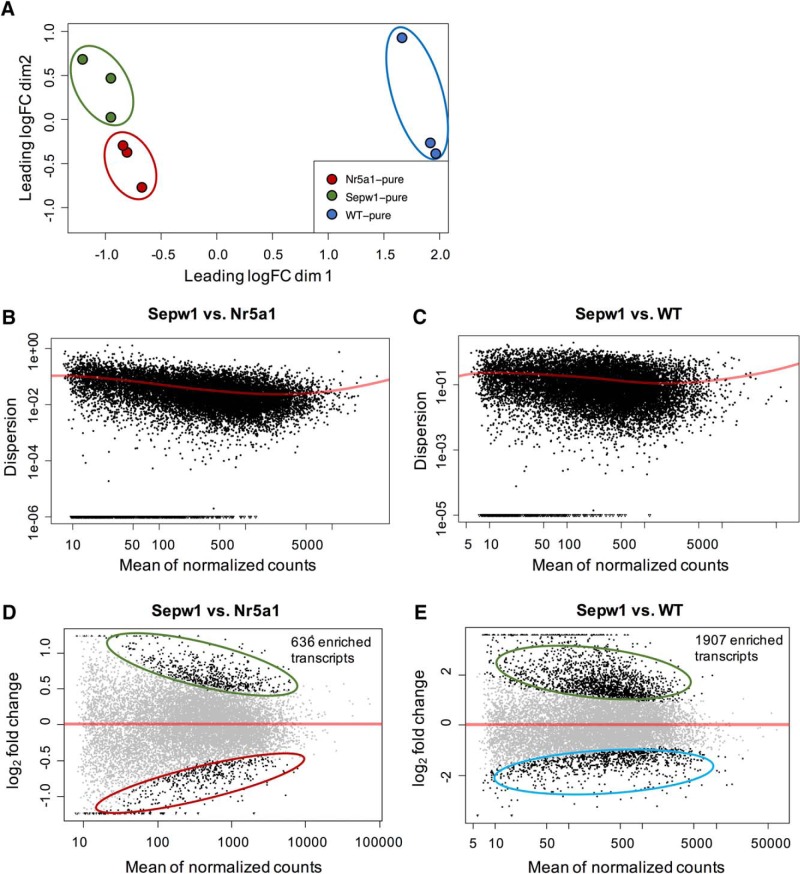
NMDS clustering of sample types and differential expression analysis. ***A***, Multiple dimensional scaling plot showing clustering of samples with the first dimension representing leading fold change distances (root mean square of the 500 largest fold changes between pairs of samples; Chen et al., 2014; Huber et al., 2015; Law et al., 2016) between Sepw1-pure, Nr5a1-pure, and WT-pure RNA sample types (filtered and normalized gene counts). ***B***, ***C***, DESeq-generated graphs showing the estimated dispersion values and fitted curves produced using filtered count data (Anders and Huber, 2012) for the Sepw1-pure to Nr5a1-pure (***B***) or Sepw1-pure to WT-pure (***C***) comparisons. ***D***, ***E***, Differentially expressed genes after DESeq analysis of Sepw1-pure samples compared to either Nr5a1-pure (***D***) or WT-pure sample types (***E***).

To approximate expression differences between samples, we generated a multiple dimensional scaling plot using the limma package in R (Ritchie et al., 2015; Law et al., 2016), which revealed the three sample types formed distinct clusters (Fig. 2*A*). We then performed pairwise comparisons between Sepw1-pure and WT-pure or Sepw1-pure and Nr5a1-pure sample types in DESeq (Anders and Huber, 2012) to determine genes with differential expression. The dispersion values for the filtered count data and fitted curve calculated for the negative binomial statistical model used in DESeq (Anders and Huber, 2012), are shown in Figure 2*B* (Sepw1 vs Nr5a1) and 2C (Sepw1 vs WT). Genes with DESeq calculated adjusted *p* > 0.1 were considered differentially expressed between sample types (Fig. 2*D*,*E*).

We next analyzed the success of the Sepw1-WT and Sepw1-Nr5a1 comparisons by identifying whether the Sepw1-gene enrichments obtained via DESeq differential expression analysis met experimental expectations. We expected to see an enrichment of genes found in upper cortical layers when Sepw1-pure sample types were compared to WT-pure or Nr5a1-pure sample types. Upper cortical layer-enriched genes can be thought of as our “signal” for this experiment, and genes expressed in lower cortical layers can be thought of as “noise.” For an unbiased description of layer-enriched genes, we used an online transcriptomic atlas of mouse neocortical layers published in Belgard et al. (2011). Although this database was created using adult rather than P12 mice, layer-defining gene expression appears to be relatively consistent after the first postnatal week when neurons have largely finished migrating (Ignacio et al., 1995). With a few exceptions (e.g., *Pou3f1*), we found similar expression patterns for Sepw1-enriched genes at both P14 and in adult mice after examining Allen Brain Atlas *in situs* at both time points (Fig. 5*B*).

**Figure 3.**
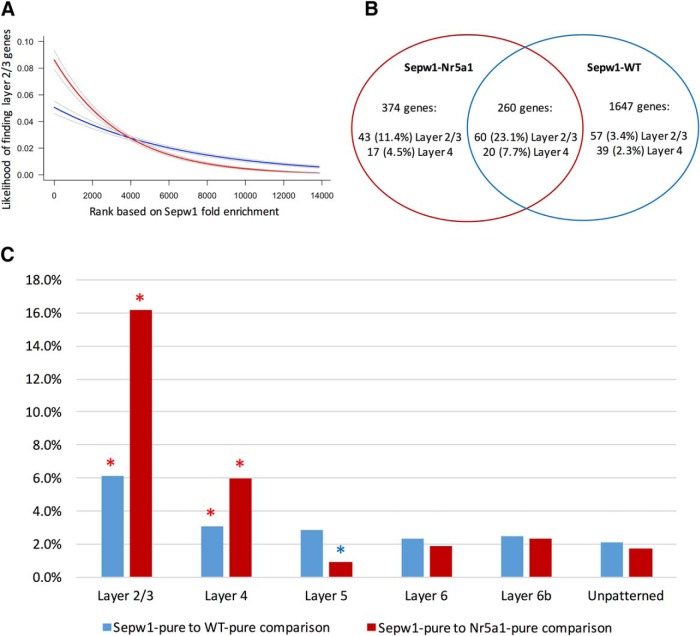
Differences in composition of Sepw1-enriched genes identified using Sepw1-Nr5a1 or Sepw1-WT comparisons. ***A***, Logistic regression analysis shows the likelihood of finding layer 2/3-enriched genes (Belgard et al., 2011) as a function of the fold change in transcript expression associated with Sepw1-enrichment. Fold changes were used to rank genes based on the level of Sepw1-enrichment, with the highest Sepw1-fold enrichment given a rank of 1 and decreasing fold enrichments given progressively higher ranks. Using both Nr5a1 (red) and WT (blue) comparisons, the likelihood of finding layer 2/3 genes is significantly greater with higher Sepw1 fold enrichment (logistic regression, *p* < 2e-16 for both WT and Nr5a1 comparisons). ***B***, Venn diagram showing the number and proportion of genes that are upper layer enriched when Sepw1-pure samples are compared to WT-pure or Nr5a1-pure samples. ***C***, Genes found in layer 2/3 are highly represented among Sepw1-enriched genes. The percentage of Sepw1-enriched genes found using Nr5a1-pure or WT-pure comparisons that overlap with database layer enriched genes, is shown. Numbers of overlapping genes falling outside the upper (red) or lower (blue) 95% confidence limits of the mean (derived from resampled distributions indicated in Table 1-1) are marked with an asterisk.

Comparing Sepw1-pure samples to WT-pure samples yielded a significant enrichment of genes expressed highly in upper cortical layers (significant differences from resampled estimates; Table 1-1; Table 1; Fig. 3). However, many nonspecific gene enrichments were also produced using this comparison. Of the 1907 Sepw1-enriched genes identified (Table 1-2), only a small fraction show layer 2/3-specific expression, presumably a consequence of the large differences in gene expression found between a heterogeneous populations of cortical cells and the subset of upper layer excitatory neurons we labeled (Chatzi et al., 2016). Transcripts found in all neuronal cells, or with high rates of transcription and/or low transcript half-lives might also be enriched using the WT-pure comparison (see Discussion). We conclude that the Sepw1-pure to WT-pure comparison has limited utility in identifying cell type-enriched gene expression.

**Table 1.** Number and classification of transcripts enriched in Sepw1-pure compared to WT-pure or Nr5a1-pure RNA sample types

Comparison	Sepw1-WT	Sepw1-Nr5a1
Number enriched (*p*adj = 0.1)	1907	634
Layer 2/3	117*	103*
Layer 4	59*	37*
Layer 5	55	6*
Layer 6	45	12
Layer 6b	47	15
Unpatterned	40	11

Genes significantly enriched in various cortical layers (400 top enriched genes per layer from an online database published in Belgard et al., 2011) were compared to the genes most significantly enriched (after DESeq differential expression analysis) in Sepw1-pure samples when compared to WT-pure (Table 1-2), or Nr5a1-pure (Table 1-3) sample types. Experimentally derived numbers of overlapping genes falling outside the upper (layer 2/3 and layer 4) or lower (layer 5) 95% confidence limits of the mean (derived from resampled distributions indicated in Table 1-1) are marked with an asterisk. Raw gene counts for all samples are listed in Table 1-4.

10.1523/ENEURO.0181-17.2017.t1-1 Table 1-1 Resampling estimates for number of database genes overlapping with DESeq Sepw1-enriched genes. List of standard deviations, estimates, CIs, and *p* values calculated using a resampling method to estimate expected values for the number of layer-enriched database genes that overlap with either 1907 (Sepw1-WT) or 634 (Sepw1-Nr5a1) randomly selected genes. The number of genes randomly selected was equal to the number of Sepw1-enriched genes identified using either WT-pure or Nr5a1-pure sample type comparisons; *p* values were calculated using resampled estimates and experimental values. Asterisks indicate experimental values that fell outside the upper or lower 95% confidence limits. Download Table 1-1, DOCX file.

10.1523/ENEURO.0181-17.2017.t1-2 Table 1-2 List of DESeq-enriched genes from the Sepw1-pure to WT-pure comparison. A total of 1907 enriched genes from the Sepw1-WT comparison, showing DESeq statistics for enrichment. Download Table 1-2, XLSX file.

10.1523/ENEURO.0181-17.2017.t1-3 Table 1-3 List of DESeq-enriched genes from the Sepw1-pure to Nr5a1-pure comparison. A total of 634 enriched genes from the Sepw1-Nr5a1 comparison, showing DESeq statistics for enrichment. Download Table 1-3, XLSX file.

10.1523/ENEURO.0181-17.2017.t1-4 Table 1-4 Raw gene counts for Sepw1-pure, Nr5a1-pure, and WT-pure sample types. List of raw gene counts for each sample counted by htseq. All protein coding genes in the mouse transcriptome identified using Ensembl biomart are represented. Download Table 1-4, XLSX file.

To obtain a greater enrichment of transcripts specific to layer 2/3, we compared Sepw1-pure samples to purified RNA from the *Nr5a1-cre* line, which sparsely labels cells in layer 4, but little if any in layer 2/3 at P12 (Fig. 1*D*,*F*,*H*). We hypothesized that the comparison of *Sepw1-cre* labeled RNAs found in layers 2/3 and 4 and *Nr5a1-cre*-labeled RNAs found only in layer 4, would yield an enrichment of layer 2/3-specific transcripts. As expected, comparing highly similar neuronal cell types (layer 2/3 vs layer 4) produced fewer differentially expressed genes (634 Sepw1-enriched transcripts; Fig. 2*D*) than comparing less similar Sepw1-pure and WT-pure sample types (1907 Sepw1-enriched transcripts; Fig. 2*E*). In addition, a much greater proportion of the Sepw1-enriched transcripts from the Sepw1-Nr5a1 comparison (as compared to the Sepw1-WT comparison) overlapped with genes expressed in layer 2/3 neurons (Table 1; Fig. 3*C*).

We examined the probability of finding layer 2/3-enriched transcripts using either Sepw1-pure/Nr5a1-pure or Sepw1-pure/WT-pure comparisons by logistic regression analysis (Fig. 3*A*). Both comparisons demonstrated an increased probability of finding a layer 2/3-enriched gene with increased Sepw1-fold enrichment, and this relationship is more pronounced when using Nr5a1-pure as a comparison (Fig. 3*A*). The Venn diagram in Figure 3*B* shows that the highest percentage (23.1%) of layer 2/3 genes are found among the overlapping 260 genes enriched using both comparisons, with 11.4% and 3.4% classified as layer 2/3 enriched among the genes found exclusively using the Nr5a1-pure or WT-pure comparison, respectively. Because the list of Sepw1-enriched genes identified using the Nr5a1-pure comparison contained the greatest percentage of layer 2/3 transcripts (Fig. 3*C*), we conclude that this dataset would be the most useful for finding genes important for the development of visual cortex layer 2/3 (Table 1-3). It should be noted, however, that there are many possible explanations for why different Sepw1-gene enrichments are observed when using either Nr5a1-pure or WT-pure as a comparison (see Discussion).

### GO analysis of layer 2/3 gene expression reveals genes associated with neuron projection development

**Figure 4.**
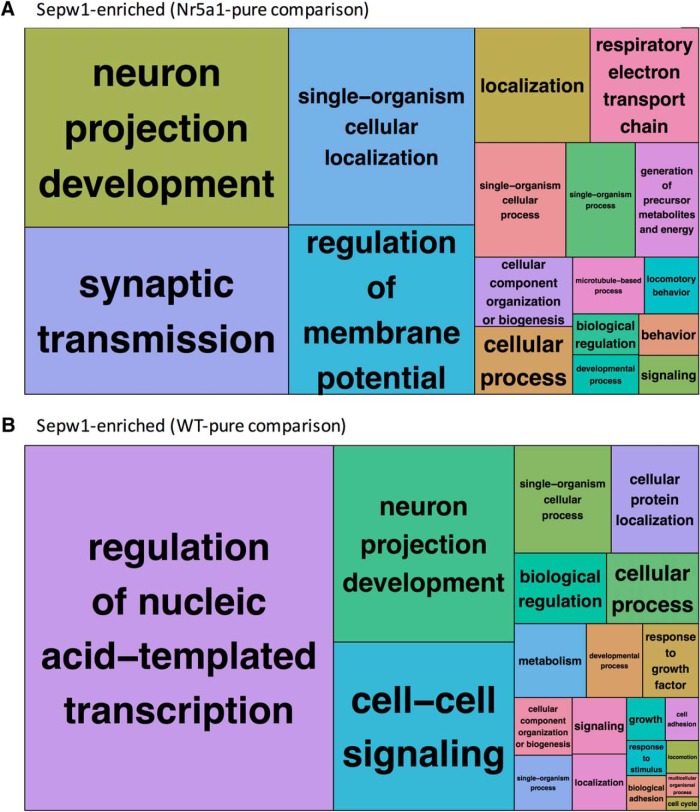
REVIGO GO tree-maps showing differences in Sepw1-enriched gene classifications using WT-pure or Nr5a1-pure comparisons. ***A***, REVIGO tree-map showing GO clusters formed using Sepw1-enriched transcripts when Nr5a1-pure is used as a comparison; *p* values and GO classifications used to create this tree map are shown in Figure 4-1. ***B***, REVIGO tree-map showing GO clusters formed using Sepw1-enriched transcripts when WT-pure is used as a comparison; *p* values and gene-ontology classifications used to create this tree map are shown in Figure 4-2.

To determine the potential functions of genes identified as enriched using both Sepw1-WT and Sepw1-Nr5a1 comparisons, we used GOTermFinder (http://go.princeton.edu/cgi-bin/GOTermFinder) to obtain GO terms that were significantly overrepresented among Sepw1-enriched transcripts. GO terms were summarized using REVIGO (http://revigo.irb.hr/), a tool designed to remove redundant terms and visualize broad categories of gene function (Boyle et al., 2004; Supek et al., 2011). The REVIGO tree-maps in Figure 4*A*,*B* show that Sepw1-pure genes are over-represented for “neuron projection development,” and differences are observed in overall gene-classification when either Nr5a1-pure (Fig. 4*A*) or WT-pure (Fig. 4*B*) is used as a comparison (see Discussion).

10.1523/ENEURO.0181-17.2017.f4-1 Figure 4-1 Table of REVIGO reduced GO terms and associated *p* values, Sepw1-WT comparison. Table containing information on the REVIGO reduced GO terms from GO-TermFinder and their associated *p* values for enrichment. The GO terms listed were further grouped to form the tree-map categories shown in Figure 4*A*. Download Figure 4-1, XLS file.

10.1523/ENEURO.0181-17.2017.f4-2 Figure 4-2 Table of REVIGO reduced GO terms and associated *p* values, Sepw1-Nr5a1 comparison. Table containing information on the REVIGO reduced GO terms from GO-TermFinder and their associated *p* values for enrichment. The GO terms listed were further grouped to form the tree-map categories shown in Figure 4*B*. Download Figure 4-2, XLS file.

### Validation of layer 2/3 enrichment of Sepw1-pure RNAs by *in situ* hybridization

**Figure 5.**
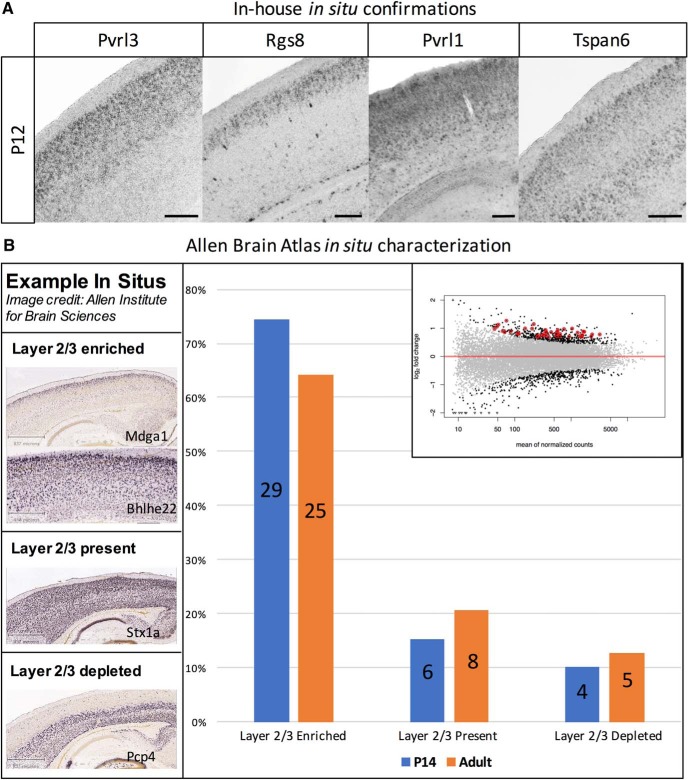
*In situ* confirmations of Sepw1-enriched genes. ***A***, In house *in situ* hybridizations to four genes of interest, *Pvrl3*, *Rgs8*, *Pvrl1*, and *Tspan6*, demonstrated upper layer specific or enriched expression at P12 (scale bar: 200 μm). *In situs* to three additional genes showed expression throughout cortex (Fig. 5-1). ***B***, Approximately 70% of select Sepw1-enriched genes show layer 2/3 enrichment, confirmed using *in situ* data. The percentage of Sepw1-enriched genes (Sepw1-pure to Nr5a1-pure comparison) found in various expression categories, based on Allen Brain Atlas *in situ* data, is shown. Of the top 300 genes found to be Sepw1-enriched, 44 had existing *in situ* data at P14 found at allenbrain.org (Developing Mouse Brain; Allen Institute for Brain Science, 2008). These *in situs* were classified manually as: layer 2/3 enriched, layer 2/3 present (no enrichment), or layer 2/3 depleted (examples found in left panel). Unclear database *in situs* were excluded from this analysis. Most genes were found to be either enriched or present in cortical layer 2/3 neurons. While the expression patterns of a few of these genes changed from P14 (blue) to adult (orange), most showed similar expression patterns over development (Mouse Brain; Allen Institute for Brain Science, 2004). Genes included in the analysis are circled in the DESeq differential expression plot in red (upper right), and highlighted red in Extended Table 1-3.

We performed *in situ* hybridization experiments to determine the laminar expression of Sepw1-pure RNAs found enriched using the Nr5a1-pure comparison. We determined the expression patterns of seven genes of interest directly by performing *in situ* hybridizations at P12. Genes of interest were chosen based on presence in the GO categories, biological adhesion (*Tspan6*, *Pvrl3*, *Pvrl1*, *Speg*), biological regulation (*Rgs8*), or synapse formation (*Sez6l2*, *Frmpd4*; Boyle et al., 2004; Mi et al., 2013 ; Mi et al., 2016). We found that four of these seven genes displayed enriched expression in layer 2/3 at P12 (*Pvrl3*, *Rgs8*, *Pvrl1*, *Tspan6*; Fig. 5*A*). The remaining three genes were expressed in all cortical layers (*Frmpd4*, *Sez6l2*, *Speg*; Fig. 5-1).

10.1523/ENEURO.0181-17.2017.f5-1 Figure 5-1 *In situ* hybridizations showing pan-neuronal expression of three genes of interest. *In situ* hybridization showing pan-neuronal expression of three genes shown to be enriched in Sepw1-pure samples when compared to Nr5a1-pure samples at P12. These three genes were chosen for analysis based on GO categorization as cell-adhesion molecules or involvement in synapse formation (scale bar: 200 μm). Download Figure 5-1, TIFF file.

We additionally used *in situ* data available at the Allen Brain Atlas website (Allen Institute for Brain Science, 2004, 2008) to determine the expression patterns of Sepw1-pure genes. Although Allen Brain Atlas (Developing Mouse Brain; Allen Institute for Brain Science, 2008) did not have expression data at P12, the stage our experiments were done, *in situ* data at P14 was available for 44 of the top 300 Sepw1-enriched genes (compared to Nr5a1-pure, 44 genes labeled in red in Table 1-3). To determine the expected expression patterns of Sepw1-pure genes, we carefully considered our Sepw1-Nr5a1 comparison, which should yield an enrichment of genes expressed highly in layer 2/3 as compared to layer 4. This leaves a variety of expected layer 2/3-enriched expression patterns, including genes with expression darkest in layer 2/3 and 5 (*Tspan6*), and genes expressed throughout cortex but darkest in layer 2/3 (*Bhlhe22*). For this reason, genes with layer 2/3-enriched expression that were not necessarily layer 2/3 “specific” were considered experimental successes and counted as enriched in our analysis. Manual classification of the expression patterns of Sepw1-pure genes revealed that the majority (∼70%) were enriched in cortical layer 2/3, and that the expression patterns of these genes did not dramatically change between P14 and adulthood (Fig. 5*B*).

### Demonstrating 4TU crosses the BBB using a newly developed *TetO-UPRT* transgenic mouse

**Figure 6.**
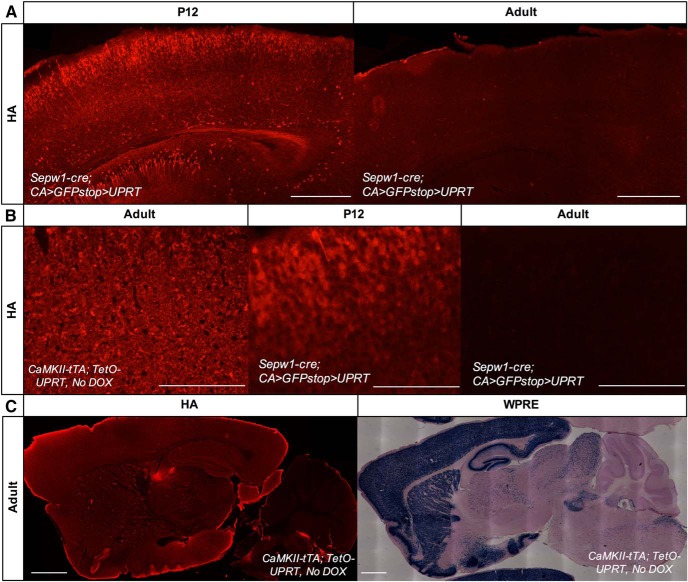
Adult neuronal expression of UPRT using a newly developed *TetO-UPRT* mouse. ***A***, Immunohistochemistry for UPRT using an HA antibody in P12 and adult *Sepw1-cre; CA>GFPstop>HA-UPRT* animals (scale bar: 500 μm) demonstrates silencing of the *CA>GFPstop>HA-UPRT* transgene in adult neurons. The images of visual cortex shown were processed identically (10× objective, 250-ms exposure, Photoshop-adjusted brightness: -30, contrast: 100). ***B***, A transgenic cross between the newly developed *TetO-UPRT* transgene (off DOX; Fig. 6-1) and a *CaMKII-tTA* mouse, drove high UPRT expression in adult neurons (immunohistochemistry for UPRT using an HA antibody). Adult neuronal expression of UPRT in the *CaMKII-tTA; TetO-UPRT* cross was similar to that seen in a *Sepw1-cre; CA>GFPstop>HA-UPRT* cross at P12. Images shown were processed identically (20× objective, scale bar: 200 μm, 14-ms exposure, Photoshop-adjusted contrast: 70). ***C***, Immunohistochemistry using an HA antibody demonstrates expression of UPRT in CaMKII-positive adult neurons in a *CaMKII-tTA; TetO-UPRT* cross (scale bar: 1000 μm, 5× objective, exposure: 150 ms, Photoshop-adjusted brightness: 30, contrast: 100). *In situ* to WPRE RNA in a *CaMKII-tTA; TetO-UPRT* mouse demonstrates strong neuronal expression of the transgene (scale bar: 1000 μm, 10× objective). Both immunostaining for HA-UPRT and *in situ* to WPRE also demonstrated leaky expression of UPRT in animals carrying only the *TetO-UPRT* transgene not crossed to a tTA line (Fig. 6-2).

While it is clear from the data presented here that 4TU injected subcutaneously can pass the BBB at P12, it is unknown if this is the case in adult animals. We found it was not possible to test whether 4TU was passing the BBB in adults using the *CA>GFPstop>HA-UPRT* mouse, since this mouse experiences transgene silencing in adult neurons (Fig. 6*A*).

10.1523/ENEURO.0181-17.2017.f6-1 Figure 6-1 Development of a *TetO-UPRT* mouse with neuronal expression responsive to DOX. ***A***, Figure of the TetO-UPRT transgene. ***B***, HA-staining for UPRT before and after DOX administration (scale bar: 1000 μm, 5× objective). Both images were taken with an exposure time of 150 ms and were adjusted identically for brightness and contrast (Photoshop-adjusted brightness: 30, contrast: 100). DAPI is also shown to highlight underlying cell structure. Mice were placed on high DOX (2 μg/ml administered in 1% sucrose water) for one week. The transgene was DOX responsive, with a substantial reduction in UPRT protein expression after one week of DOX administration, although a small amount of leaky expression remained. Placing mice on high DOX from birth may help with leaky expression. Download Figure 6-1, TIFF file.

10.1523/ENEURO.0181-17.2017.f6-2 Figure 6-2 Leaky expression of UPRT in a single positive *TetO-UPRT* mouse. There is some leaky UPRT expression even when no tTA is present in a single positive *TetO-UPRT* transgenic mouse. This leaky expression is mostly limited to hippocampus, although the cerebellum and thalamus also appear weakly positive for the UPRT protein (2.5× objective, scale bar: 1000 μm). Primary visual cortex (V1) and areas of potential leaky expression are labeled. Red arrow: hippocampus; orange arrow: cerebellum; green arrow: thalamus. Download Figure 6-2, TIFF file.

To determine if 4TU injected subcutaneously can reach adult neurons, we developed a new *TetO-UPRT* mouse. When this line is crossed to a cell type-specific tTA or rtTA line, UPRT expression is induced depending on the absence (tTA) or presence (rtTA) of tetracycline or doxycycline (DOX). The transgene itself incorporates a tetracycline operator (tetO) sequence driving the expression of a hemagglutinin (HA) epitope-tagged UPRT gene followed by a WPRE, intended to increase levels of gene expression (subsequently called *TetO-UPRT*; Fig. 6-1). When crossed to the *CaMKII-tTA* neuronal line, we observed high transgene expression in adult neurons (Fig. 6*C*), and treatment of this mouse with DOX reduced transgene expression (Fig. 6-1). However, we detected “leaky” neuronal expression that persisted after DOX administration (Fig. 6-1) and was also present in animals carrying only the *TetO-UPRT* transgene (Fig. 6-2). For this reason, we conclude that this mouse should only be used cautiously for future TU-tagging experiments. However, since even the leaky expression was neuronal, we were able to use the line to determine if 4TU crossed the BBB.

For this experiment, 4TU was injected subcutaneously into two WT and two *TetO-UPRT; CaMKII-tTA* adult mice, hippocampi were dissected, and RNA was extracted, biotinylated and streptavidin purified. After purification, the levels of tagged RNA from the transgenic cross far exceeded that of the WT mice (Table 2). In addition, this experiment was performed using a *TetO-UPRT* mouse not crossed to anything to determine the amount of RNA tagged by leaky neuronal UPRT expression. Consistent with UPRT expression levels (Fig. 6; Fig. 6-2), the amount of RNA purified from single positive *TetO-UPRT* hippocampal tissue fell between the quantities obtained using WT or double positive *CaMKII-tTA; TetO-UPRT* hippocampal tissue (Table 2). The increased levels of purified RNA obtained from hippocampi with neuronal UPRT expression (compared to UPRT negative tissue) indicate that 4TU injected subcutaneously in adult mice is passing the BBB and reaching neurons.

**Table 2.** Percentage yield of thiol-labeled RNA from hippocampal neurons after subcutaneous administration of 4TU

Sample type	Age	Tissue	Percent yield
*TetO-UPRT; CaMKII-tTA*	P50	Hippocampus	3.90%
*TetO-UPRT; CaMKII-tTA*	P90	Hippocampus	3.20%
*TetO-UPRT*	P120	Hippocampus	2.00%
*TetO-UPRT*	P120	Hippocampus	2.00%
WT	P50	Hippocampus	0.07%
WT	P90	Hippocampus	0.10%

*CaMKII-tTA; TetO-UPRT* double positive mice, *TetO-UPRT* single positive mice, or WT control mice were injected subcutaneously with 4TU and hippocampus was removed 5 h later. After RNA extraction and streptavidin purification, the amount of tagged RNA obtained was much higher from hippocampal samples expressing UPRT in neurons (*CaMKII-tTA* driven neuronal expression and leaky neuronal expression in single positive *TetO-UPRT* mice; Fig. 6-2).

## Discussion

The TU-tagging method described here can be used to isolate cell type-specific RNA *in vivo* through the targeted expression of the enzyme UPRT. This technique may be particularly useful for identifying nascent RNAs in neuronal cell types, since the long axonal/dendritic processes that define these cell types can be damaged or removed during physical isolation processes such as laser capture microdissection or cell sorting. There are multiple ways to target UPRT expression to a specific cell type. A recent paper used virus to express UPRT in two different neuronal types (Chatzi et al., 2016). Although virus is an effective way to control the number and type of UPRT expressing cells, working with virus requires special safety considerations and manipulations, and is capable of inducing an immune response *in vivo* (Lowenstein et al., 2007). Virus injection also requires invasive surgery, which is not always practical depending on age. Using transgenic mice to target UPRT expression to specific cell types requires no such manipulations, and can be an excellent alternative to viral methods depending on the experimental question. Here, we demonstrate the first successful application of TU-tagging in mouse neurons using transgenic mice, and are also the first to demonstrate 4TU injected subcutaneously can pass the BBB.

In this experiment, we used TU-tagging to identify genes enriched in layer 2/3 of murine visual cortex around eye opening. To thiol-label nascent RNAs in cell types enriched in layer 2/3 of developing visual cortex, we crossed *CA>GFPstop>HA-UPRT* and *Sepw1-cre* transgenic mice. To identify genes enriched in layer 2/3, we compared RNA purified from *Sepw1-cre* labeled neurons (Sepw1-pure), to RNA purified from: (1) a sparse subset of layer 4 neurons labeled using an *Nr5a1-cre; CA>GFPstop>HA-UPRT* transgenic cross (Nr5a1-pure), or (2) WT cortical tissue not expressing UPRT (WT-pure). To maximize our yields of tagged RNA, we used a modified TU-tagging protocol, which included a 5- to 6-h wait time between 4TU injection and visual cortex dissection (we have found that the amount of purified RNA from a ubiquitously expressing UPRT mouse P6 brain peaks between 4 and 6 h after 4TU injection; L. Gay and C. Q. D., unpublished data), and poly-A selection of unfragmented RNA after streptavidin purification (Chatzi et al., 2016). This TU-tagging workflow allowed the preparation of small amounts of RNA for sequencing and made possible the comparison of purified RNA from a neuronal cell type to extremely low yields of WT purified RNA. In our hands, the amount of RNA purified from both neuronal UPRT+ and WT tissue was insufficient to process for sequencing using the previously published TU-tagging protocol (Gay et al., 2014; data not shown).

By directly comparing samples containing *Sepw1-cre* labeled RNAs to Nr5a1-pure and WT-pure sample types, we successfully isolated genes enriched in layer 2/3 of visual cortex at P12. Although the highest percentage enrichment of layer 2/3 genes was found using the Sepw1-Nr5a1 comparison (Fig. 3; Table 1), the Sepw1-WT comparison may provide more expansive information about the differences between layer 2/3 neurons and the rest of cortex. Due to the higher variability between Sepw1-pure and WT-pure sample types, genes with low fold enrichments had overall higher DESeq adjusted *p* values using this comparison than with the Sepw1-Nr5a1 comparison (lowest fold changes of enriched genes with an adjusted *p* < 0.1; Sepw1-WT = 1.9; Sepw1-Nr5a1 = 1.36). Therefore, it appears the Nr5a1-comparison allowed the detection of genes with subtler Sepw1-enrichment, which may not be represented using the WT-comparison (Fig. 3*B*, 374 exclusive Sepw1-Nr5a1 gene enrichments). A GO analysis of Sepw1-enriched transcripts revealed that many were involved in neuron projection development, suggesting that our selection of the P12 time point allowed the identification of genes involved in synapse formation (Fig. 4). We conclude that the comparison of two streptavidin purified sample types can help to isolate signal in a TU-tagging experiment, and comparing two highly similar sample types, such as Sepw1-pure and Nr5a1-pure, may help narrow results to those specifically enriched in a particular cell type.

While our method was successful in isolating cell type-enriched genes, the direct comparison of purified RNAs isolated using different Cre lines may yield biases related to litter and strain differences. This is a potential confound for all RiboTag, TRAP, and TU-tagging studies where cell type-enriched RNAs were isolated using multiple mouse strains. An unbiased correction for strain differences would require access to the same specific cell types across strains, and is beyond the scope of this paper. Since the *CA>GFPstop>HA-UPRT* strain would have provided 50% of the genetic make-up for crosses containing the *Sepw1-cre* and *Nr5a1-cre* lines, genetic differences between these crosses should have been minimal. The WT samples were also composed of mice sharing at least 50% genetic similarity to the *Sepw1-cre* mice. Future experiments comparing two different Cre lines may further reduce strain differences by crossing transgenic lines to an inbred strain over multiple generations. Although it is possible that strain differences resulted in false positives in our enrichment data, it is unlikely that we would have seen the pattern of layer 2/3 enrichment observed if we were not isolating true differences between cell types.

When designing a TU-tagging experiment it is important to consider many variables that may affect whether final gene enrichments reflect true differences in expression between cell types. While the original TU-tagging method (Gay et al., 2013, 2014) called for the direct comparison of streptavidin-purified RNA to unpurified total RNA, the comparison of two RNA samples that experienced different types of processing could yield nonspecific gene enrichments. Since RNA transcripts can vary in their susceptibility to degradation, whole sample changes in transcript composition can occur with heavy processing of RNA during purification. Bias can be introduced at various stages in sample preparation: early, due to transcript to transcript variability in the efficiency of thiol-labeling and conjugation to HDPD biotin, and later, during cDNA synthesis of poly-A selected RNAs and Nextera library preparation (Cui et al., 2010; Head et al., 2014; Lahens et al., 2014; Duffy et al., 2015). Since sample preparation alone can produce differential gene expression unrelated to underlying biological processes, we avoided comparisons between streptavidin-purified RNA samples and unpurified RNA in this study.

It is also possible for RNA thiol-labeled outside the UPRT expressing cell type to contaminate a streptavidin purified sample. There are a few biochemical pathways in mammals that can, at a much lower rate, carry out the same 4TU to 4-thiouridine conversion performed by UPRT, which may lead to a small amount of nonspecific thiol-labeling of RNAs (Tallafuss et al., 2014). The use of transgenic mice may also contribute to noise if low levels of nonspecific UPRT transcription occurs outside of cell types expressing Cre or tTA (leaky UPRT expression; Fig. 6-2). The 4-thiouridine made by UPRT+ cells may also diffuse into neighboring UPRT-negative cells, leading to thiol-labeled RNA in these cells. This has been observed in coculture experiments (G. Zhang and R. Goodman, Vollum Institute, personal communication), and may be amplified by developmental processes such as apoptosis or synaptic pruning *in vivo*. Here, we attempted to reduce noise effects caused by nonspecific labeling and 4-thiouridine diffusion by comparing similarly processed sample types and limiting our wait time between 4TU injection and tissue harvest to 5–6 h.

Varying wait times between 4TU injection and tissue harvest can also influence noise that arises from transcript to transcript differences in transcription rate and half-life. In our experiments, WT-pure samples are likely largely composed of contaminating unlabeled RNAs derived from total RNA, containing many fewer newly transcribed RNAs than the Sepw1-pure sample types. When there is a large discrepancy in the levels of newly transcribed RNA between compared sample types, as is the case with the Sepw1-WT comparison, many gene enrichments or depletions may simply reflect transcriptional dynamics. Comparing streptavidin purified RNA samples to total RNA samples is analogous to a ratio of newly transcribed RNA/total RNA, a ratio which has also been used to infer transcript half-lives (Dölken et al., 2008). Consequently, transcriptional dynamics alone can produce large enrichments for genes with high transcription rates (large numerator) and high rates of decay (small denominator), depending on the time allowed for transcription. Interestingly, “regulation of nucleotide templated transcription” was the most highly represented cluster from the REVIGO analysis of Sepw1-enriched genes from the Sepw1-WT comparison using a 5-h wait time (genes involved in “regulation of transcription” have some of the shortest half-lives; Sharova et al., 2009; Fig. 4). Using longer wait periods may instead select for transcripts with long half-lives, such as extracellular matrix, cytoskeletal, metabolism, and protein synthesis related genes (Sharova et al., 2009). In this study, the Sepw1-Nr5a1 comparison appeared to largely eliminate this type of noise (Fig. 4).

One of the most important considerations when designing a TU-tagging experiment, is the selection of sample type comparisons. It is important to note that the specificity of expression obtained (cell type enriched vs cell type specific) is contingent on the comparison used. When using Cre lines that drive sparse expression in specific cell types, a significant portion of the streptavidin-purified samples derived from these lines may contain noise from unlabeled or mislabeled RNAs. For this reason, the Cre line for which expression data are desired should have equal or greater UPRT expression (and therefore labeled RNAs) than the Cre line used as a comparison. For example, we would not recommend using a pan-neuronal Cre line as a comparison for a sparse neuronal Cre line, since background from mislabeled or unlabeled RNAs may be enriched in the sample derived from the sparse Cre line. Contaminating RNAs not specific to the UPRT expressing cell type should be less influential when using Cre lines with dense expression (increased signal-to-noise), and may eventually be eliminated with improvements to RNA labeling and purification protocols (Duffy et al., 2015; Hida et al., 2017).

With the appropriate selection of a sample type comparison, the TU-tagging method described here can identify newly transcribed genes in sparse cell types *in vivo*. While some protocol alterations made here may eliminate a few possible benefits of the method (poly-A selection prevents the isolation of microRNAs or long noncoding RNAs, and removal of the fragmentation step may decrease overall levels of gene enrichment), by selecting a streptavidin purified comparison, the technique becomes significantly more sensitive to changes in gene expression. Current improvements to the chemistry of biotinylation (Duffy et al., 2015), the development of new labeling and purification protocols (Hida et al., 2017), new transgenic lines for targeted UPRT expression, and a better understanding of the types of noise to expect, should together help make the TU-tagging technique more accessible for future transcriptional profiling experiments.

## Synthesis

Reviewing Editor: Mike Fainzilber, Weizmann Institute of Science Decisions are customarily a result of the Reviewing Editor and the peer reviewers coming together and discussing their recommendations until a consensus is reached. When revisions are invited, a fact-based synthesis statement explaining their decision and outlining what is needed to prepare a revision will be listed below.

The reviewer's concerns were largely addressed, but there are still a few issues with the revision that need to be done to make the manuscript more clear to the reader and better emphasize the data presented.

The work does bring an interesting approach and getting the data out will benefit other investigators considering the TU-tagging approach. We appreciate the authors raising a number of caveats for the approach and that they cannot directly compare unlabeled RNA harvested from WT to the purified TU-tagged RNA. It would be worthwhile for the authors to point out what could be directly compared, as the SepW1 to Nr5a1 comparison is quite conservative (e.g., a pan-neuronal cre-driver). Also it would be useful to specifically point out that the comparison used filtered out only the lower layer cortical mRNAs, so that they may be including some mRNAs that are shared between layer 2/3 layer 5/6 as their data shows (e.g., Tspan6 in Fig 5B). In general, the authors need to clearly stress the point that the method is not absolute and is driven by choice of cre-drivers and their specific expression.

Furthermore, the authors should qualify the statement in line 463-465 to indicate that the RNA yield was not sufficient ‘in our hands’ or something to that effect. Other labs may be able to get that to work and the sensitivity of NGS instrumentation and methods continues to advance.

The authors should switch the order of Fig 5 and description to better emphasize their own data. The in house in situs are good data and the authors' data rather than a reference database should come first with the Allen work confirming their work. Tspan6 does not appear to be quite as Layer 2/3 enriched as the authors suggest - there is substantial signal in the lower cortical layers. Finally, it is not clear what the authors are showing from the Allen Institute images. What mRNAs or proteins are displayed in those images?

Minor points:

The tables and figures referring to layers would be clearer if the authors used the same nomenclature as in the text. Frankly, they do not save much room by abbreviating ‘Layer’ as ‘L’, so suggest that they revise Figures and Tables to reflect that (and use 2/3 as in text rather than ‘23’).

Line 45, ‘fluorescently labeled’ should read ‘GFP-tagged’ or ‘fluorescent reporter-tagged’. Nomenclature used suggests a direct fluorescently conjugation of the ribosomal protein.

Line 343, suggest to use and/or for ‘rates of transcription and low transcript half-lives’.
