# Supplementary material for: TU-Tagging: A Method for Identifying Layer-Enriched Neuronal Genes in Developing Mouse Visual Cortex
Source: eNeuro. 2017 Oct 4;4(5):ENEURO.0181-17.2017. doi: 10.1523/ENEURO.0181-17.2017 (PMC5659240; doi:10.1523/ENEURO.0181-17.2017)
Supplement: Table 1-1 [file enu005172418so3.docx]

**Extended Table**

**Table 1-1. Resampling estimates for number of database genes overlapping with DESeq Sepw1-enriched genes.**

| **Tissue type** | **Standard deviation** | **Estimate** | **Lower_CI** | **Upper_CI** | **Experimental value** | **P-value** |
| --- | --- | --- | --- | --- | --- | --- |
| **Sepw1-Pure to WT-Pure Comparison, 1907 Enriched Genes** | | | | | | |
| Layer 2/3 | 5.83 | 39.93 | 28.26 | 51.59 | 117* | < 0.001 |
| Layer 4 | 5.13 | 31.72 | 21.46 | 41.98 | 59* | < 0.001 |
| Layer 5 | 6.47 | 50.47 | 37.53 | 63.41 | 55 | 0.429 |
| Layer 6 | 5.87 | 42.17 | 30.43 | 53.92 | 45 | 0.613 |
| Layer 6b | 5.84 | 43.51 | 31.84 | 55.18 | 47 | 0.534 |
| Unpatterned | 6.53 | 47.22 | 34.15 | 60.28 | 40 | 0.26 |
| **Sepw1-Pure to Nr5a1-Pure Comparison, 634 Sepw1-Enriched Genes** | | | | | | |
| Layer 2/3 | 3.63 | 13.35 | 6.07 | 20.61 | 103* | < 0.001 |
| Layer 4 | 3.18 | 10.48 | 4.11 | 16.84 | 37* | < 0.001 |
| Layer 5 | 3.85 | 16.6 | 8.90 | 24.31 | 6* | 0.002 |
| Layer 6 | 3.58 | 14.02 | 6.85 | 21.18 | 12 | 0.501 |
| Layer 6b | 3.71 | 14.48 | 7.06 | 21.89 | 15 | 0.786 |
| Unpatterned | 3.86 | 15.66 | 7.93 | 23.38 | 11 | 0.180 |
