# Supplementary figures and images for: TU-Tagging: A Method for Identifying Layer-Enriched Neuronal Genes in Developing Mouse Visual Cortex
Source: eNeuro. 2017 Oct 4;4(5):ENEURO.0181-17.2017. doi: 10.1523/ENEURO.0181-17.2017 (PMC5659240; doi:10.1523/ENEURO.0181-17.2017)

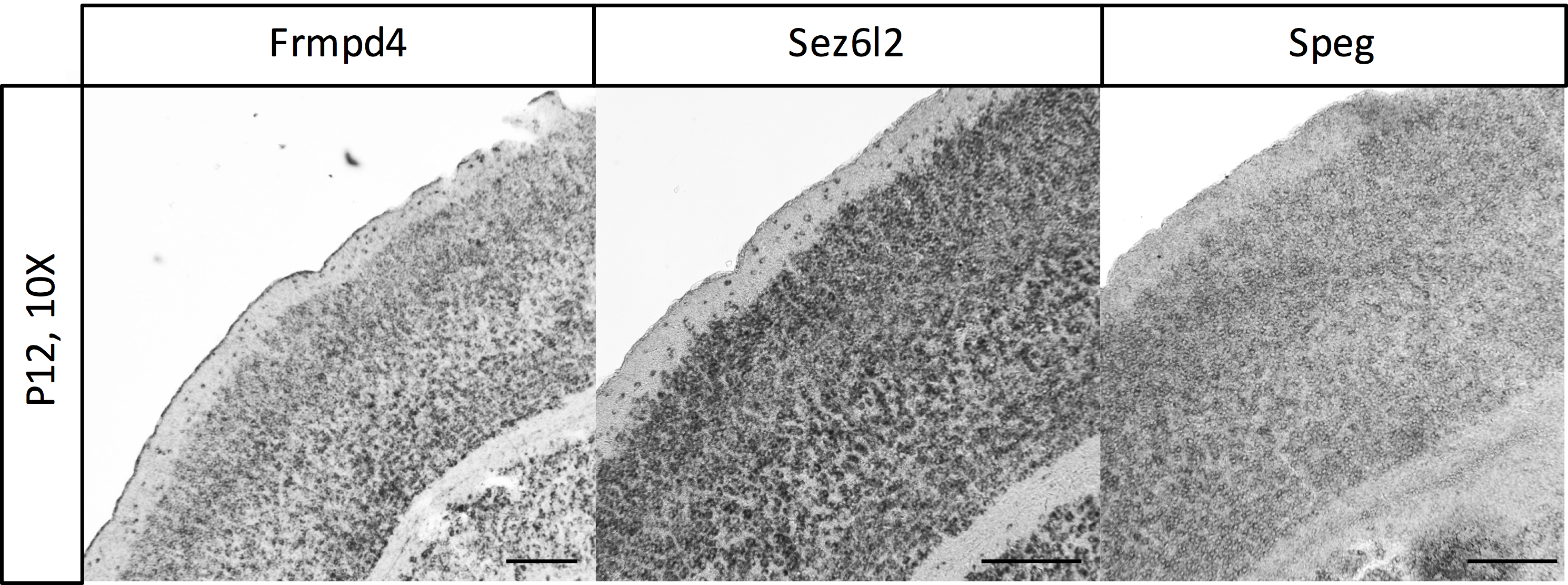

Supplement: Figure 5-1 [file enu005172418so7.tif]

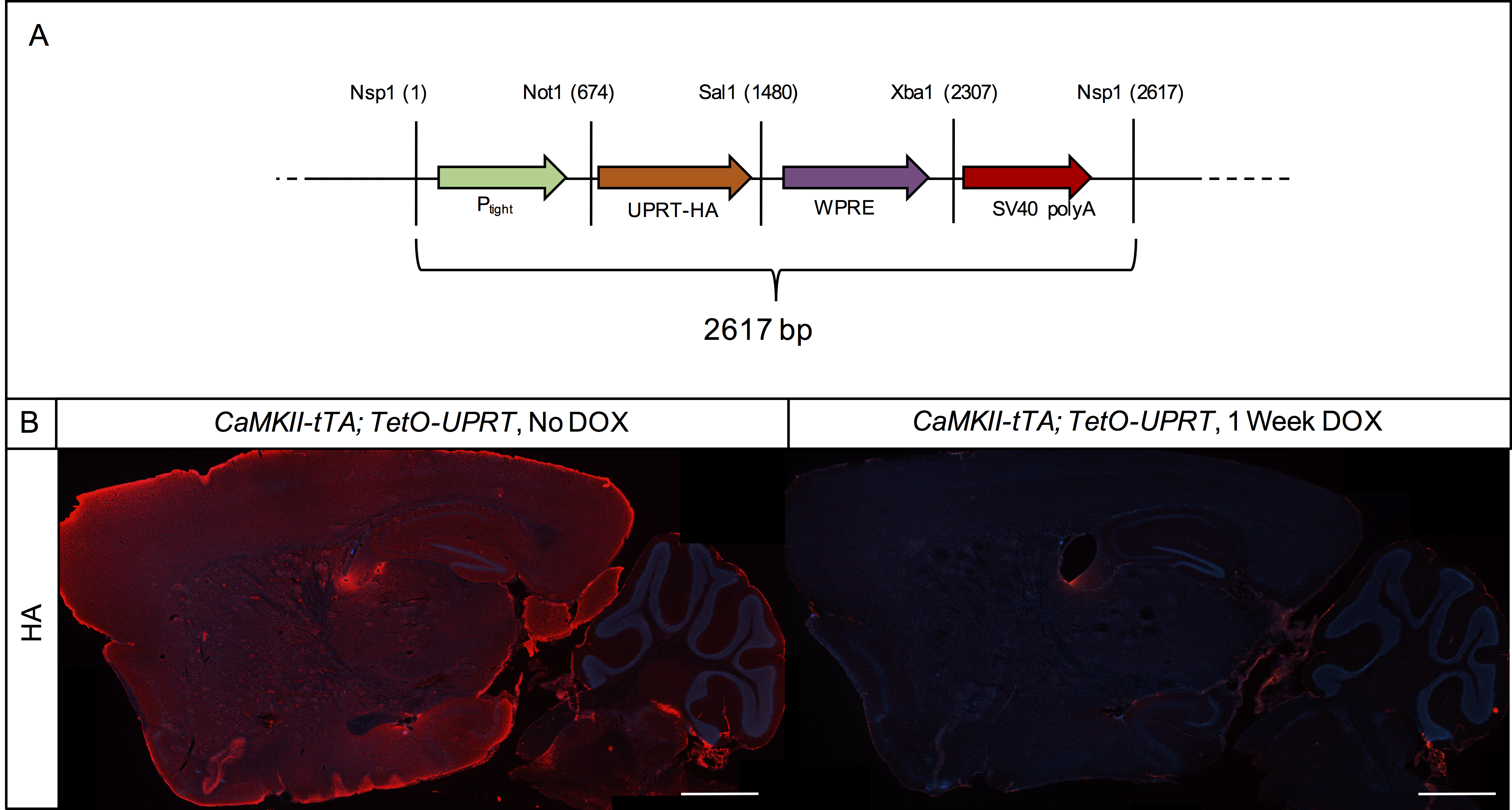

Supplement: Figure 6-1 [file enu005172418so8.tif]

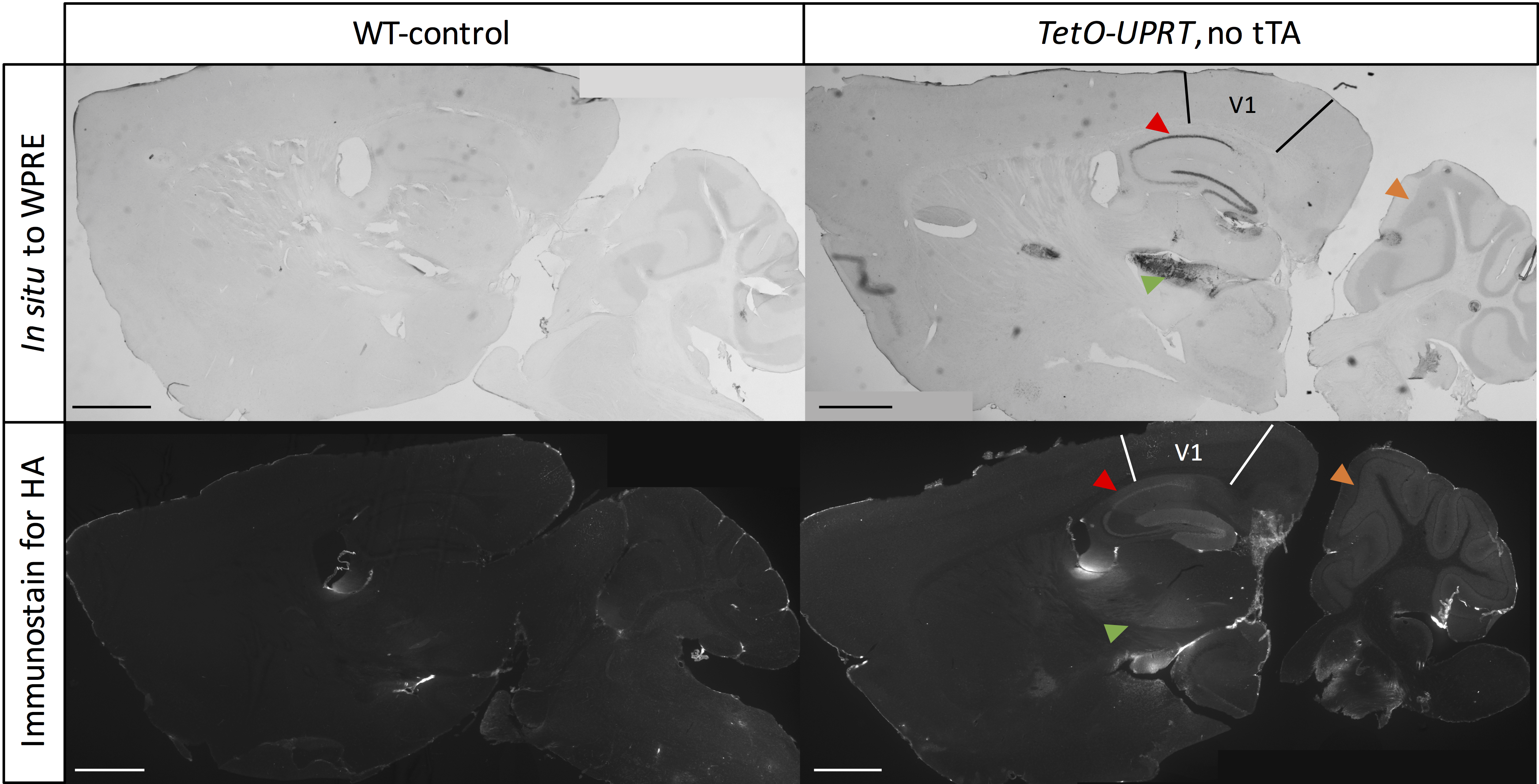

Supplement: Figure 6-2 [file enu005172418so9.tif]
